# MNet-10: A robust shallow convolutional neural network model performing ablation study on medical images assessing the effectiveness of applying optimal data augmentation technique

**DOI:** 10.3389/fmed.2022.924979

**Published:** 2022-08-16

**Authors:** Sidratul Montaha, Sami Azam, A. K. M. Rakibul Haque Rafid, Md. Zahid Hasan, Asif Karim, Khan Md. Hasib, Shobhit K. Patel, Mirjam Jonkman, Zubaer Ibna Mannan

**Affiliations:** ^1^Department of Computer Science and Engineering, Daffodil International University, Dhaka, Bangladesh; ^2^College of Engineering, IT & Environment, Charles Darwin University, Darwin, NT, Australia; ^3^Department of Computer Science and Engineering, Ahsanullah University of Science and Technology, Dhaka, Bangladesh; ^4^Department of Computer Engineering, Marwadi University, Rajkot, India; ^5^Department of Smart Computing, Kyungdong University – Global Campus, Sokcho-si, South Korea

**Keywords:** medical image, ablation study, geometric augmentation, photometric augmentation, shallow CNN, deep learning models

## Abstract

Interpretation of medical images with a computer-aided diagnosis (CAD) system is arduous because of the complex structure of cancerous lesions in different imaging modalities, high degree of resemblance between inter-classes, presence of dissimilar characteristics in intra-classes, scarcity of medical data, and presence of artifacts and noises. In this study, these challenges are addressed by developing a shallow convolutional neural network (CNN) model with optimal configuration performing ablation study by altering layer structure and hyper-parameters and utilizing a suitable augmentation technique. Eight medical datasets with different modalities are investigated where the proposed model, named MNet-10, with low computational complexity is able to yield optimal performance across all datasets. The impact of photometric and geometric augmentation techniques on different datasets is also evaluated. We selected the mammogram dataset to proceed with the ablation study for being one of the most challenging imaging modalities. Before generating the model, the dataset is augmented using the two approaches. A base CNN model is constructed first and applied to both the augmented and non-augmented mammogram datasets where the highest accuracy is obtained with the photometric dataset. Therefore, the architecture and hyper-parameters of the model are determined by performing an ablation study on the base model using the mammogram photometric dataset. Afterward, the robustness of the network and the impact of different augmentation techniques are assessed by training the model with the rest of the seven datasets. We obtain a test accuracy of 97.34% on the mammogram, 98.43% on the skin cancer, 99.54% on the brain tumor magnetic resonance imaging (MRI), 97.29% on the COVID chest X-ray, 96.31% on the tympanic membrane, 99.82% on the chest computed tomography (CT) scan, and 98.75% on the breast cancer ultrasound datasets by photometric augmentation and 96.76% on the breast cancer microscopic biopsy dataset by geometric augmentation. Moreover, some elastic deformation augmentation methods are explored with the proposed model using all the datasets to evaluate their effectiveness. Finally, VGG16, InceptionV3, and ResNet50 were trained on the best-performing augmented datasets, and their performance consistency was compared with that of the MNet-10 model. The findings may aid future researchers in medical data analysis involving ablation studies and augmentation techniques.

## Introduction

In today’s world, cancer is an alarming threat to global health. In 2020, around 19.3 million new cancer cases and approximately 10 million new cancer deaths were recorded worldwide (1). By 2040, global cancer cases are estimated to be increased by 47%, resulting in 28.4 million new cancer cases (1). Early detection of cancer and well-timed and effective treatment increase chances of survival leading to reduced mortality rates. If diagnosed in a primary stage, the only treatment necessary may be a simple surgery (2, 3). In many countries, however, the number of clinicians is not sufficient for the number of patients (4). Due to the growing number of patients, it can be unmanageable for a doctor or a specialist to diagnose the disease in the early stage without any automated system. As interpretation of many medical images can lead to fatigue of clinical experts, computer-aided interventions may assist them in reducing the strain associated with high-performance interpretation (5). With the development of CNN-based applications in medical image analysis (6), clinical specialists benefit from CAD by utilizing outputs of a computerized analysis to identify lesions, evaluate the existence and extent of diseases, and improve the accuracy and reliability of diagnosis by decreasing false negative rates. Hence, incorporating CAD approaches into medical diagnostic systems lessens the workload and pressure of doctors, thereby increasing early detection (7). Currently, medical imaging procedures, for instance, mammography, ultrasound, X-ray, dermoscopy, CT scan, and MRI, are used for diagnosis and identification of diseases (8). However, the information and semantics of a picture can greatly vary with different images having different visual characteristics and appearances based on the disease and modality. In several cases, variability in shape, size, characteristics, the intensity of lesions, and distinctive imaging characteristics, even within the same modality, causes diagnostic challenges even to medical experts. Often, the intensity range of a cancerous region may be similar to surrounding healthy tissues. Due to the presence of noise and artifacts and poor resolution of images, simpler machine learning approaches tend to yield poor performance with manually extracted features (9). To overcome these challenges, deep learning models have been employed in medical image classification, segmentation, and lesion detection over the past few decades with noteworthy advances (4). Rather than extracting and feeding features manually to a network, deep learning deals directly with an image dataset by discovering useful representations in an automated manner. CNNs can acquire more complex features by focusing on a potential irregular region (9).

These challenges are compounded by insufficient number of training images or imbalance in the number of images for different classes. As a solution, data augmentation is a commonly used technique that aids in improving the performance of CAD systems by generating new images. However, even after applying data augmentation or other techniques, overfitting may not always be prevented, resulting in poor performance. A possible cause might be not applying the most suitable augmentation techniques given the characteristic of the dataset. The approaches employed for a particular task cannot be expected to perform with optimal accuracy on different datasets or modalities (10). Deep convolutional neural networks (DCNNs) have made great progress in various computer vision-related image classification tasks. However, because of having a complex network structure with a large number of layers, DCNNs often require extensive computing and memory resources and training time. Moreover, as the total number of parameters of DCNNs is high, they require a large number of training data to yield a good performance without overfitting.

For this study, eight medical datasets for various diseases and modalities are used, including a mammogram dataset, a skin cancer dermoscopy dataset, a COVID chest X-ray dataset, a brain tumor MRI dataset, a chest CT-scan dataset, a breast cancer ultrasound image dataset, a breast cancer microscopic image dataset, and a tympanic membrane dataset. We propose a high-accuracy robust CNN model with a shallow architecture and performing ablation study to achieve a satisfactory performance across the eight medical datasets. The model is named MNet-10, as the depth of the architecture is 10 weighted layers, and is constructed to classify medical images. The performance of CNN models greatly varies with alteration of layer architecture, the filter number, and size, as well as different hyper-parameters. A particular model might perform with high accuracy for a particular dataset while providing poor performance and causing overfitting issues for another dataset with a different imaging modality. Hence, developing a specific model with high accuracy for several medical image datasets with different imaging modalities is quite challenging. Moreover, no image pre-processing step is employed on the datasets before feeding them into the model. In interpretation of raw images, abnormality detection is more difficult for a CNN model because of complex hidden characteristics of a region of interest (ROI).

Each of the datasets used for this research has a different nature, characteristics, and challenges. We have studied all the datasets and identified the main challenges and characteristics of the images that need to be addressed. The challenges of medical images with deep learning are

(1) In most cases, when overfitting occurs because of a limited number of images, a network can learn and remember the features of training instances but cannot apply this learning to an unobserved dataset.
(2) For images where it is vital to preserve the geometrical location and orientation of an irregular region, classification accuracy might drop without employing suitable augmentation techniques.
(3) Due to the presence of artifacts, detection of abnormality using raw medical images is another substantial challenge in developing a robust CNN classification model for the medical domain.
(4) Deep learning requires an input dataset to be well balanced, but datasets often contain a highly imbalanced number of images in different classes. Because of this inconsistency, the resulting model tends to perform well in classes with more data and poor for classes with fewer data. Several approaches can be carried out to address the data imbalance issue depending on the problem to be solved. The most widely used techniques include data augmentation, generation of synthetic images with generative models (11), and cutting of the number of images from classes containing the highest number of pictures.
(5) In intra-class classification problems, intra-class similarities, same class dissimilarities, limited color intensity distribution, and intensity similarity between cancerous lesions and surrounding tissues often occur and lead to high misclassification rate.
(6) In some datasets, the size of images is unequal. As deep learning requires an equal size for all images, the images need to be resized to a particular size, and useful information might be lost for some of the pictures. Regarding resizing of images, the original size of the images should be considered first while setting the parameter value of resizing. If the image size of a dataset is found very large or irregular, the parameter should be set in such a way that the size is not reduced drastically and useful information can be preserved.

As a result of these concerns, the optimal configuration of the hyper-parameters of the architecture can only be set up after an extensive assessment process that can deal with all of the challenges described above. Our proposed network is developed by determining suitable layer architecture, parameters, and hyper-parameter values based on highest accuracy.

In studies on medical imaging, usually, a particular augmentation approach aids to improve the model’s performance for a particular imaging modality where it might cause poor performance for the other modality. Therefore, it is crucial to ascertain a suitable augmentation technique based on the characteristics of a dataset. In this research, the experiment is carried out using non-augmentation, photometric augmentation, and geometric augmentation techniques for all eight datasets to explore which approach yields the optimal outcome for which dataset. It is found that the performance varies depending on different augmentation approaches for the different datasets.

**FIGURE 1:**
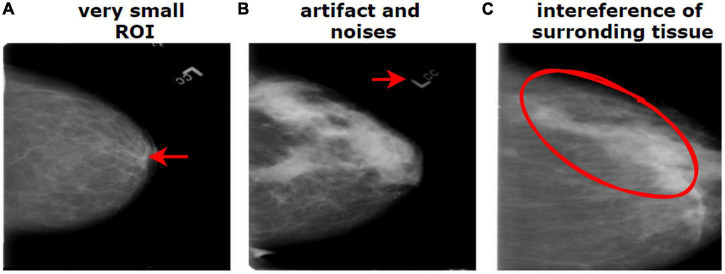
Challenges of medical datasets showing breast mammography. **(A)** Very small ROI and **(B)** presence of artifacts. **(B)** The dissimilarity between the same class and **(C)** similarity between different classes.

The breast cancer mammography dataset we have used in this study contains most of the challenges described above including limited number of images, presence of unwanted regions, hidden ROIs, interference of surrounding dense tissue, similarities between different classes, and dissimilarities within the same class. Figure 1 shows a mammography example of the challenges of medical datasets.

To develop the proposed MNet-10 model employing an ablation study, the breast cancer mammography dataset is used as all the challenges of interpreting medical images are found in the mammography dataset. We hypothesize that if a model can address the challenges in the mammography dataset, it might also provide good performance across other datasets. The results suggest that for all the eight datasets, the model is able to yield a good performance where in most cases photometric augmentation yields better performance than geometric augmentation.

Along with the photometric and geometric augmentation methods, elastic deformation is also conducted as a data augmentation technique to observe the performance. By elastic deformation, the shape, geometry, and size of an object can be altered even in a complex way (12).

To summarize, we examined, by extensive ablation study and data augmentation, how an optimal model can be generated using effective ablation study approaches and suitable data augmentation techniques where the resulting model performs with high accuracy even for different medical datasets with different modalities. The findings and approaches of this study might assist future medical researchers in understanding the importance of choosing optimal augmentation techniques and developing a robust model after an ablation study. This research can be an effective approach for developing a robust model having optimal configuration and data augmentation technique with the purpose of interpreting medical images with different imaging modalities.

## Literature review

Over the past decades, astonishing progress in medical imaging has occurred with discoveries of hidden features of diseases and their progression. To the best of our knowledge, no research similar to ours, using different medical imaging modalities to classify diseases while exploring several augmentation schemes, has been conducted so far.

Kumar et al. (10) proposed an ensemble technique to categorize the modality of medical images using multiple fine-tuned CNNs to extract optimized features of different imaging modalities. They experimented with several hyper-parameters and parameter values to find the optimal architecture and achieved a satisfactory outcome. A data augmentation approach was used with a 10-fold augmentation system, which included cropping and flipping methods. Ashraf et al. (13) attempted to classify different medical images for several body organs by employing a fine-tuning scheme to a pre-trained deep CNN model. The authors generated a combined dataset of 12 classes of human body organs (e.g., chest, breast, colon, etc.) utilizing various available online medical image databases. The average overall accuracy of the proposed approach was around 98%. However, no augmentation scheme was used in their research. Zhang et al. (14) used four medical datasets of different categories such as skin lesions, MRI, and CT to classify different modalities. They highlighted intra-class similarities and dissimilarities for different abnormalities and imaging modalities. Their model synergic deep learning (SDL) was proposed by employing multiple DCNNs that are able to learn from each other simultaneously.

As augmentation techniques, geometric and photometric augmentation approaches are often described in medical image research. Elgendi et al. (15) studied the influence of geometric augmentations introduced in various current research studies for identifying COVID-19. The performance of 17 deep learning models on three COVID-19 chest x-ray datasets was compared before and after applying different geometric augmentation techniques. The results showed that the elimination of geometrical augmentation methods increased the Matthews correlation coefficient (MCC) for the 17 algorithms. However, only geometric augmentation was explored in this study. Another study (16) for detecting breast masses employed the Digital Database for Screening Mammography (DDSM) and explored eight augmentation schemes, such as Gaussian noise, Gaussian blur, flipping, and rotation, and compared the outcomes. After training the VGG16 model, their highest accuracy, using a Gaussian filter and rotation methods, was 88%, and their lowest accuracy, after inducing noise, was 66%. Taylor et al. (17) examined several common data augmentation methods, including geometric and photometric, to find which approaches are most suitable for a particular dataset. They evaluated various data augmentation procedures using a simple CNN architecture on the Caltech101 dataset comprising a total of 9,144 images in 102 classes. They achieved the highest accuracy, 79.10%, using a cropping scheme. However, no description of the 102 classes is given in the article, and no ablation study was carried out while developing the CNN model. Mikołajczyk et al. (18) investigated several ways of data transformations, namely, rotation, crop, zoom, photometric schemes, histogram-based approaches, style transfer, and generative adversarial networks for their image classification tasks. Using a VGG16 model, the augmentation methods were evaluated with three medical datasets: skin cancer melanomas, breast histopathological images, and breast cancer MRI scans. No clear description of the analysis and construction of the proposed model was found in the article. Falconi et al. (19) used the CBIS-DDSM mammography dataset to detect abnormalities in the form of binary classification problems by employing transfer learning and fine-tuning approaches. They performed data augmentation by employing the geometric method and the photometric method and applying histogram equalization on images. Milton et al. (20) used the ISIC skin cancer dermoscopy dataset to classify cancers using the transfer learning approach. They introduced a variety of data augmentation techniques combining geometric and photometric methods. Sajjad et al. (21) classified brain tumor MRIs using a fine-tuned VGG16 model and data augmentation techniques including four geometric alterations and four noise invariance schemes.

## Dataset description

As stated previously, eight different medical imaging modalities are experimented with in this research. The ultrasound image dataset of breast cancer is a publicly available dataset consisting of three classes from Kaggle (22), and the dataset contains a total of 780 images where 133 are found in the normal class, 440 in the benign class, and 207 in the malignant class. The size of the images is 500 pixels × 500 pixels.

We employ the mammogram dataset provided by the Curated Breast Imaging Subset of The Digital Database for Screening Mammography (CBIS-DDSM) database from Kaggle (23) and consist of four classes. The dataset contains a total of 1,459 images, where 398 are found in the benign calcification class, 417 in the benign mass class, 300 in the malignant calcifications class, and the remaining 344 in the malignant mass class. In this dataset, all the mammograms are 224 pixels × 224 pixels.

A collection of chest X-rays with four classes from the COVID-19 Radiography Database available in Kaggle (24) is also used. This dataset contains 3,616 images of patients positive for COVID-19, with 1,345 viral pneumonia, 6,012 lung opacity (non-COVID lung infection), and 10,192 normal cases in a grayscale format. All of the images in this dataset are 299 pixels × 299 pixels.

A collection of skin cancer images are analyzed in this research from the International Skin Imaging Collaboration 2020 (ISIC 2020) challenge, collected from Kaggle, separated into two classes (25). In this dataset, the benign class contains 1,800 images, and the malignant class contains 1,497 images. All of the images in this collection are 224 pixels × 224 pixels and in an RGB format.

The tympanic membrane, from the Cardiotocography (CTG) Analysis database (26) contains a total of 956 otoscopic images. The dataset contains a total of nine classes, of which four, the Normal class (535 images), Earwax (140 images), Acute otitis media (119 images), and chronic suppurative otitis media (63 images), are included in this study as they form the majority of the images. The remaining five classes, Otitis external, Ear ventilation, Foreign bodies in the ear, Pseudo membranes, and tympanosclerosis, consist of a total of 99 images, with less than 50 images in each class. As they contain insufficient samples, the five classes are not included in this study. All the images are 500 pixels × 500 pixels.

Three different classes of MRI scans are studied in this study containing brain tumor samples along with data from healthy patients that are collected from Kaggle (27). The dataset contains four classes with 926 images consisting of glioma tumors, 937 images of meningioma tumors, 901 images of pituitary tumors, and 500 images without tumors.

Microscopic biopsy images of benign and malignant breast cancers from the Breast Cancer Histopathological Database (BreakHis), collected from Kaggle (28), are also included in this study. A total of 1,693 images in two classes are analyzed where 547 images of benign tumors and 1,146 images of malignant tumors are considered.

The CT scan images from a chest cancer dataset collected from Kaggle (29) are employed in this study containing a total of 613 CT scan images classified into four classes: adenocarcinoma (195 images), large cell carcinoma (115 images), squamous cell carcinoma (155 images), and, finally, normal (148 images).

Anyone can access and share the datasets and employ them in their research study because all the datasets are publicly available to contribute to research.

**FIGURE 2:**
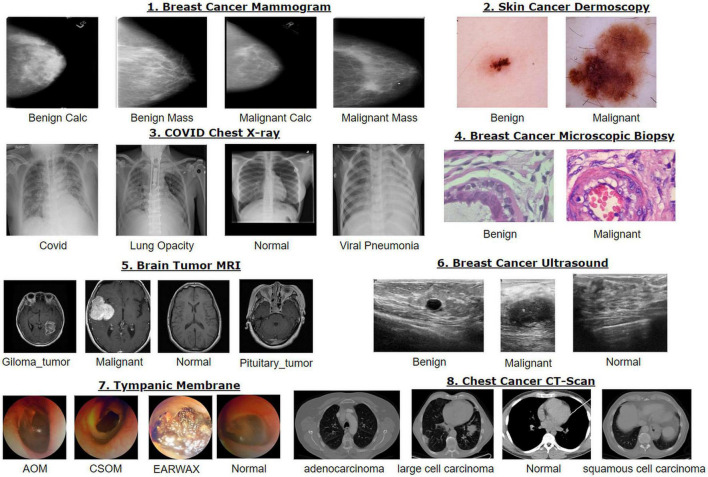
Datasets used in this research.

A sample of the eight modalities and their classes that are used in this research are illustrated in Figure 2.

## Proposed methodology

**FIGURE 3:**
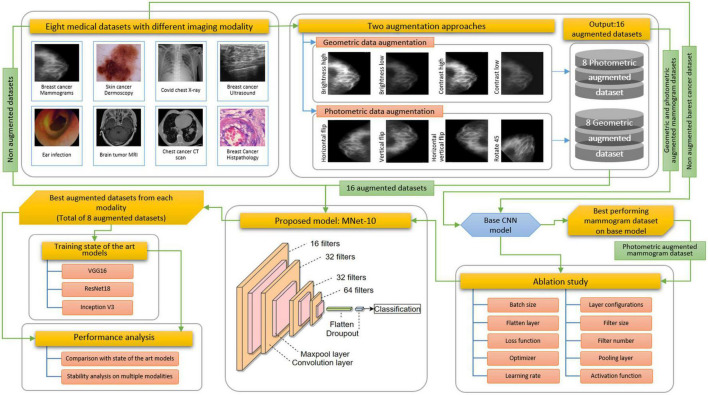
Overview of the proposed methodology.

Challenges resulting from the nature of the datasets described above are commonly addressed following three steps: employing appropriate image pre-processing techniques, data augmentation, and a robust deep learning model with suitable hyper-parameters. In medical image analysis, publicly available datasets are often found to have a limited number of images for training deep learning models. Besides, complex lesion structure, useful hidden patterns, and pixel information make the medical image analysis task challenging and error-prone. Any technique, algorithm, or model should be selected based on the characteristics of the dataset, and after investigating the essential pixel information remains intact. As data augmentation is commonly performed in computer vision tasks especially in medical imaging, applying a suitable technique might improve accuracy. Two augmentation approaches are explored to show how a similar technique’s performance varies for different datasets. A particular technique might not be suitable for all datasets. However, as no image pre-processing techniques are employed in this study, the network should be developed in such a way that all the challenges described above can be addressed resulting in a good performance while using raw images. Figure 3 illustrates the complete process of this study.

As described in Section “Dataset description,” images from the different datasets have unequal pixel sizes. As a CNN requires equal size images to train, pictures of all the datasets are first resized to a 224 pixels × 224 pixels size. As upscaling the size of an image might result in a blurry and distorted image, the smallest image size among all the datasets is considered the standard input size for the proposed CNN model. In this regard, among the datasets that are used in this research, 224 pixels × 224 pixels are found to be the lowest pixel size. Therefore, this has been chosen as the standard input image size of the proposed CNN model. However, the image size of all the datasets is not very large and closer to this size. Therefore, resizing the images to 224 pixels × 224 pixels has proven to be an efficient method that shows no signs of distortion. Hence, the performance of the proposed model is not impacted by it. Afterward, all the datasets are augmented using geometric and photometric approaches where both approaches consisted of four transformation techniques. We initially construct a base CNN model to perform an ablation study where the breast cancer mammogram dataset is chosen for this ablation study. Afterward, the base model is trained with all augmented and non-augmented mammogram datasets. The dataset with the highest accuracy is used to train the base CNN. To develop the architecture of MNet-10 with an optimal configuration, an ablation study is performed. While experimenting with different hyper-parameters and layer structures, characteristics of other datasets are also considered. Later, the model MNet-10 is applied to the other seven datasets both before and after augmentation. Our CNN-based framework gives good performance while having low computational complexity without overfitting concerns across a variety of medical image datasets with varying imaging modalities. The results suggest that in most cases, photometric augmentation has the best performance. Afterward, three often used deep learning models, namely VGG16, InceptionV3, and ResNet50 are applied with the best performing augmented datasets, and their performance are compared with the proposed architecture for a rigorous investigation of performance consistency across different modalities. According to our findings, although for some datasets an acceptable outcome is achieved, the performance of the deep learning architectures is not stable across all the eight datasets. A comprehensive discussion on why performance varies with different augmentation techniques is presented at the end of the article.

### Data augmentation

Data augmentation refers to the method of generating new images similar to the training dataset and is considered a regularization technique to prevent overfitting issues (30). Regularization techniques prevent overfitting while training models, whereas data augmentation addresses the issue at the root of the task which is the training set. Augmented data should be generated in such a way that they represent a more comprehensive set of possible data points, consequently reducing the difference between the training and validation datasets as well as any unseen testing sets. Generating new data should be conducted in such a way that pixel details remain intact, which is essential to preserve medical information. Data augmentation can be denoted as the mapping (31):


(1) ϕ:𝒮↦𝒯

where *S* represents the original dataset and *T* denotes the augmented dataset of *S*. Therefore, the artificially inflated training dataset can be stated as:


(2) $${𝒮}^{{}^{\prime }}=𝒮\cup 𝒯$$

where *𝒮*′ contains the original dataset and the corresponding alterations are represented by *T*.

We have experimented with two augmentation techniques, namely, geometric augmentation and photometric augmentation, on each of the datasets to evaluate their performance over different modalities. The two augmentation approaches comprise four photometric methods and four geometric methods. In both approaches, the number of augmentation techniques is kept the same for the number of augmented images to remain equivalent.

#### Geometric augmentation

This alteration method changes the geometry of a given image by mapping distinct pixel values to new endpoints. The fundamental structure and details contained in an original image are preserved but transformed to new points and alignment. In our study, four geometric augmentation techniques, namely, vertical flipping, horizontal flipping, rotation 90°, and rotation −90°, have been used for the dataset. Flipping, as a geometric augmentation technique, often appears as a convenient tactic for natural images, and numerous research studies have been conducted in this field (16). However, in medical imaging study, flipping including both vertical and horizontal are adopted widely across several modalities of mammogram (32, 33), dermoscopy images (34, 35), chest CT scan (36, 37), chest X-ray (15, 38), brain tumor MRI (21, 39), tympanic membrane (40, 41), breast cancer histopathology image (42, 43), and breast cancer ultrasound images (44, 45) which might be an obvious reason acquiring poor performance as the alteration may not result in clinical possible images. Though flipping a medical image such as an MRI scan would cause a scan one would almost never see in the clinical setting, it is often claimed to be an effective strategy (39, 46). Therefore, both of the techniques are explored in this research as a segment of the geometric approach to assess performance using different datasets with different modalities so that an optimal scheme can be suggested for future studies. Other classical geometric data augmentation schemes such as cropping, zooming, shearing, and scaling are not applied as some medically relevant pixel regions might be eliminated.

#### Vertical flipping

Flipping reproduces an image around its horizontal or vertical axis. In vertical flipping, an image is alternated upside down in a way that the original *x*-axis is retained and the *y*-axis is replaced. The equation can be stated as (31):


(3) $$\left[\begin{array}{c} f_{x}\\ f_{y} \end{array}\right]=\left[\begin{array}{cc} 1 & 0\\ 0 & -1 \end{array}\right]\cdot \left[\begin{array}{c} x\\ y \end{array}\right]$$

Here, *x* and *y* denote the pixel coordinates of the original image, and *f_x_* and *f_y_* represent the transformed pixel coordinates after flipping *x* and *y* along the vertical axis.

#### Horizontal flipping

In this method, the original pixel coordinates of rows and columns of an image are changed horizontally based on the formula below:


(4) $$\left[\begin{array}{c} f_{x}\\ f_{y} \end{array}\right]=\left[\begin{array}{cc} -1 & 0\\ 0 & 1 \end{array}\right]\cdot \left[\begin{array}{c} x\\ y \end{array}\right]$$

#### Rotation

The rotation process on an image is applied by rotating the original pixel coordinates with a specific angle. The formula can be represented as


(5) $$\left[\begin{array}{c} f_{x}\\ f_{y} \end{array}\right]=\left[\begin{array}{cc} \text{cos}\varphi & -\text{sin}\varphi \\ \text{sin}\varphi & \text{cos}\varphi \end{array}\right]\cdot \left[\begin{array}{c} x\\ y \end{array}\right]$$

where *f_x_* and *f_y_* are the altered new points after the rotation process with an angle on original pixel coordinates *x* and *y* of the raw image. In our experiment, the values of phi are 90 and −90°.

#### Photometric augmentation

In photometric transformations, the RGB channels of an image are altered by mapping the original pixel value (r, g, and b) to new pixel values (r’, g’, and b’), which changes pixel color intensity. This changes pixel illumination, intensity, and pigment while leaving the geometry unaffected. As described, the ROI of medical images can be challenging to detect because of complex structure and hidden characteristics; therefore, any method that may affect pixel intensity should only be selected after testing with datasets. An effective augmentation technique should increase the number of images while preserving important pixel details. Without carefully choosing the technique, instead of increasing accuracy, augmentation may lead to overfitting. However, the human eye often cannot detect the loss of necessary pixels of images, especially for medical datasets.

A solution is to derive peak signal-to-noise ratios (PSNR) for all augmentation methods as an effective quality measure comparing the original image and the transformed image. PSNR value is assessed depending on pixel intensity between two images where if intensities considerably contrast, a PSNR value of less than 20 is achieved (47). This strategy is commonly adopted in several image preprocessing tasks to find that along with the preprocessing of images, what if the intensity changes to a higher extent? In respect of the photometric augmentation technique, for some methods, variations can be drastic. For some datasets having complex, subtle, and hidden characteristics, the intensity alteration could be so dire that the processed images might not be considered clinically possible. It is undeniably true that drastically different but clinically possible augmented images would benefit a model, but the question remains how someone recognizes certainly that radically altered images are clinically possible. One might not claim assuredly that although the alteration is drastic, the clinical setting of images is not impaired as human eyes often make an error while distinguishing intensity changes. In this regard, a statistical measurement such as PSNR might be a convenient approach in terms of perceiving the degree of transformation. Augmentation techniques yielding considerably low PSNR values might result in affecting the clinical setting. Ignoring these techniques can be a superior approach when choosing augmentation methods. In this study, the aim of introducing the experiment with PSNR values is to eliminate the augmentation techniques with which the lowest PSNR values are achieved indicating higher intensity dissimilarity with the original images (48).

The photometric augmentation methods employed in this study are chosen after investigating techniques named Gaussian noise, HE, hue, saturation, altering brightness, and altering contrast. We find that for the methods of hue and saturation, no changes occur in the images, which are in a grayscale format. Therefore, for the datasets of mammogram, chest x-ray, MRI, CT scan, and ultrasound, these methods cannot be introduced as augmentation techniques. For the remaining three datasets of skin cancer dermoscopy, otoscopic images (tympanic membrane dataset), and histopathology images (breast cancer microscopic biopsy images), these methods are applied, and a PSNR value is derived. Finally, we have applied Gaussian noise, HE, altering brightness, and altering contrast to each of our datasets and selected the optimal ones based on the highest PSNR value. Table 1 shows the average PSNR value (dB) of 10 randomly chosen images for each dataset and augmentation technique.

**TABLE 1:** Peak signal-to-noise ratios values of different photometric augmentation techniques.

Augmentation technique	Skin cancer dermoscopy images	Breast cancer mammogram	Tympanic membrane otoscopic images	COVID chest X-ray images	Breast cancer ultrasound images	breast cancer microscopic biopsy images	Brain tumor MRI images	Chest CT-Scan
Hue	15.63	–	18.72	–	–	14.50	–	–
Saturation	16.48	–	14.25	–	–	15.71	–	–
Noise	13.79	9.52	12.72	12.50	13.36	12.47	10.30	9.62
HE	14.72	16.39	15.19	17.19	14.25	14.42	14.51	13.19
Brightness high	29.04	30.72	29.95	29.42	30.82	29.16	29.85	29.72
Brightness low	29.12	30.23	29.19	29.15	30.61	29.31	29.17	30.35
Contrast high	34.42	31.68	31.55	31.66	32.75	31.75	32.01	34.07
Contrast low	33.59	31.89	31.54	30.71	31.11	30.11	33.04	35.36

In Table 1, it can be observed that the highest PSNR values are recorded for the augmentation techniques, brightness high, brightness low, contrast high, and contrast low, which indicates that a new diverse image is generated without losing necessary pixel information. For the other augmentation techniques, especially for noise and HE, comparatively poor PSNR is achieved, which demonstrates high dissimilarity of pixel intensity value between the original image and the augmented image. A PSNR value < 20 is not acceptable for images (47) as it indicates important pixel distortion (49). Therefore, for these photometric augmentation methods, our proposed CNN model might yield a poor performance. Therefore, we have augmented the datasets by altering the brightness and contrast of the raw images.

The term brightness of an image represents the overall lightness or darkness of the picture. Conversely, contrast is defined as the variance of intensity between the region of interest (ROI) and background pixels existing in an image. The mathematical formula for changing brightness can be stated as


(6) b(x)=s(x)+β

Here, *s*(*x*) represents the input pixels and *b*(*x*) the output pixels after changing the brightness level. Increasing or decreasing the value of parameter β will add or subtract a constant amount to each pixel. A positive value (β > 1) will result in brightening the image, whereas a negative value (β < 1) will cause darkening.

To alter the contrast level of the pixels, the difference in brightness is raised by a multiple. The mathematical formula can be stated as:


(7) c(x)=α×s(x)

Here, *s*(*x*) refers to the pixels of the source image and *c*(*x*) to the output pixels after changing contrast.

**FIGURE 4:**
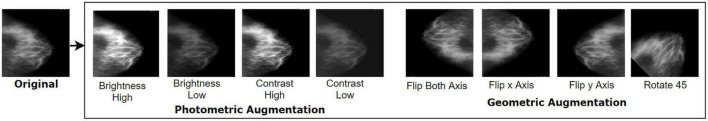
Geometric and photometric augmentation techniques.

For this photometric approach, we experimented with several beta (β) and alpha (α) values and selected α values of 1.2 and 0.8 for increasing and decreasing brightness, respectively. Likewise, the β values 1.2 and 0.8 are applied to increase and decrease, respectively, the contrast of the images. Here, the parameter value α > 1 leads to increased contrast and α < 1 to decreased contrast. Based on the above formulas, each of the datasets is augmented by employing four photometric methods: increasing brightness, reducing brightness, increasing contrast, and reducing contrast. Figure 4 shows the images after applying four geometric and four photometric augmentation techniques.

#### A brief explanation of generating augmented datasets applying different transformation techniques

As shown in Table, there are eight different types of image datasets considered to evaluate the performance of the model. The original images of every dataset are augmented using four photometric and four geometric techniques. We used different augmentation techniques directly on datasets that had almost the same number of images in each class. For the other datasets, where the number of images is highly inconsistent, we have tried to balance the number of images in each class. In this regard, only the chest X-ray and ear infection datasets are balanced. For this, a threshold for balancing the classes is determined based on the class containing the lowest number of samples. Afterward, images are cut from classes that have more samples than the threshold and brought closer to the threshold number of images. As CNNs tend to provide good results with completely balanced datasets (same number of samples in all classes), the highly imbalanced datasets are kept slightly imbalanced to perform a rigorous evaluation of the proposed model. This is achieved by starting from the threshold and gradually increasing the number of samples by a factor for classes containing the second lowest number of samples to the highest class. In this regard, classes that have similar or slightly more images from the threshold will be skipped and stay the same. In terms of the COVID-19 chest X-ray dataset, the lowest number of 1,345 images (Table 2) is found in the viral pneumonia class and considered as the threshold (1,300) for balancing the dataset, and the increasing factor is determined as 100. The number of images in the remaining classes COVID, Lung opacity, and Normal are 3,616, 6,012, and 10,192 images, respectively, which are quite greater than the threshold. After balancing the dataset, the number of images in the second lowest class (COVID) becomes 1,400, for the third lowest class (Lung opacity) 6,012, and for the highest class (Normal) 1,600. It is noticeable that in a balanced dataset, the number of images in each class is gradually increased by roughly 100 images and kept slightly inconsistent. In terms of the Tympanic membrane dataset, the number of images for classes AOM, CSOM, Earwax, and Normal is 119, 63, 140, and 533, respectively (Table 2). The fewest number of 63 images is found in the CSOM class and considered as the threshold (50) for balancing the dataset, and the increasing factor is determined as 50. Here, most of the classes are quite balanced and quite near the threshold besides the Normal class. Therefore, the number of images in the highest class (Normal) is cut down to 250 images while other classes are kept the same.

**TABLE 2:** Description of the original and augmented datasets.

Breast ultrasound image dataset				
Class	Original	Balanced	Photometric	Geometric
Benign	440	–	1760	1760
Malignant	207	–	828	828
Normal	133	–	532	532
Total	780	–	3120	3120

**COVID-19 chest X-ray image dataset**				

**Class**	**Original**	**Balanced**	**Photometric**	**Geometric**

COVID	3616	1400	5600	5600
Lung opacity	6012	1500	6000	6000
Normal	10192	1600	6400	6400
Viral pneumonia	1345	1345	5380	5380
Total	21165	5845	23380	23380

**Breast cancer mammogram image dataset**				

**Class**	**Original**	**Balanced**	**Photometric**	**Geometric**

Benign calc	398	–	1592	1592
Benign mass	417	–	1668	1668
Malignant calc	300	–	1200	1200
Malignant mass	344	–	1376	1376
Total	1459	–	5836	5836

**Skin cancer dermoscopy image dataset**				

**Class**	**Original**	**Balanced**	**Photometric**	**Geometric**

Benign	1800	–	7200	7200
Malignant	1497	–	5988	5988
Total	3297	–	13188	13188

**Tympanic membrane dataset**				

**Class**	**Original**	**Balanced**	**Photometric**	**Geometric**

AOM	119	–	476	595
CSOM	63	–	252	315
Earwax	140	–	560	700
Normal	533	250	1000	800
Total	855	527	2288	2288

**Brain tumor MRI image dataset**				

**Class**	**Original**	**Balanced**	**Photometric**	**Geometric**

Glioma tumor	926	–	3704	3704
Meningioma tumor	937	–	3748	3748
No tumor	500	–	2000	2000
Pituitary tumor	901	–	3604	3604
Total	3263	–	13056	13056

**Breast cancer microscopic biopsy image dataset**				

**Class**	**Original**	**Balanced**	**Photometric**	**Geometric**

Benign	547	–	2188	2188
Malignant	1146	–	4584	4584
Total	1693	–	6772	6772

**Breast cancer CT scan image dataset**				

**Class**	**Original**	**Balanced**	**Photometric**	**Geometric**

Left lower lobe of adenocarcinoma	195	–	780	780
Large cell carcinoma of left hilum	115	–	460	460
Normal	148	–	592	592
Squamous cell carcinoma of left hilum	155	–	620	620
Total	613	–	2452	2452

### Proposed model

The recent progress in computer-aided technology in the field of medical images, particularly in deep learning techniques, has been quite useful to medical experts for recognizing and categorizing diseases by understanding and extracting meaningful hidden patterns (50). Deep learning can extract and merge significant features related to the target abnormality detection or classification process. CAD can provide a more accurate assessment of disease progression by automated medical imaging analysis. In CNN based medical image analysis, meaningful features are learned in an automated way, which identifies meaningful patterns automatically. As stated, the main objective of this study is to develop a CNN model that is able to interpret images with (i) a limited number of training data, (ii) less computational resources and training time without compromising its performance, (iii) several medical image datasets in different domains and modalities, and (iv) yield high classification accuracy on raw images. To deal with a limited number of training data with low computational complexity and training time, a shallow CNN architecture can be an ideal approach.

Deep CNN models contain a lot of parameters that require a substantial amount of training data to perform without causing overfitting. In this regard, the scarcity of labeled medical images often hinders the performance of traditional deep CNN models (51). Conventional CNN models tend to have deeper architectures resulting in too many parameters creating issues regarding overall performance and increasing time complexity (52). This issue can be addressed by increasing the volume of the dataset utilizing image data augmentation techniques. However, too much augmentation can occasionally degrade performance in terms of parameter number (53). For small datasets containing limited samples, even with the application of the data augmentation technique four to five times, the dataset is still not sufficient enough to train a huge number of parameters of a state-of-the-art DCNN model. Furthermore, for small datasets, although the number of images is increased extensively by employing a number of augmentation techniques to meet the minimum requirement of DCNN, an optimal performance could not be achieved, as the number of original samples is still inadequate. On the other hand, reducing the number of parameters by developing a compact CNN (shallow CNN) can lower the requirement for larger datasets (54). For having lower parameters, moderate-sized datasets will much benefit from a shallow CNN model rather than a DCNN model, as containing a lower number of parameters results in better learning, which eventually produces better results. Therefore, regarding both small and large datasets, a shallow CNN is able to churn out better results utilizing a convenient number of data augmentation techniques as the number of original samples appears to be quite sufficient for training a lightweight CNN. Also, shallow CNNs tend to be faster and more efficient than deep CNNs (55), which can contribute in time complexity.

#### Base convolutional neural network model

**FIGURE 5:**
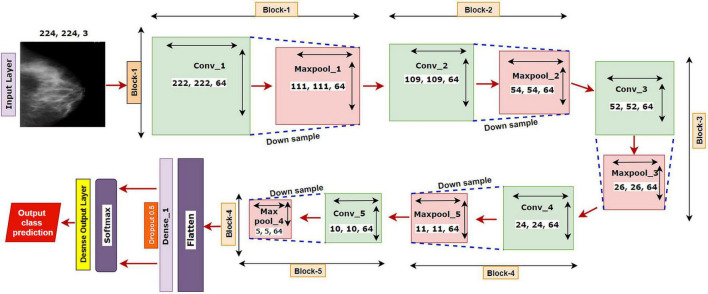
The architecture of the base model.

We have started our experiment with a base CNN model having five convolutional layers each followed by a maxpool layer. Initially, the network had 3 × 3 convolutional kernels, and the number of kernels was set to 64 for all the convolutional layers, with a dropout value of 05. “Relu” is selected as the activation function, “softmax” as the final layer activation function, and “categorical_cross entropy” as the loss function, with optimizer Adam with a learning rate of .001 and a batch size of 64. The base model is illustrated in Figure 5.

The model is run for 100 epochs with the breast cancer mammography dataset. The input shape of the images is denoted as 224 × 224 × 3, where 224 × 224 denotes the height × width, and 3 refers to the number of channels in each image (color channel in RGB format). In a convolutional layer, a dot operation of input and weight is performed that outputs the feature map using the following equation:


(8) $$h^{k}=f\left(W^{k}*x+b^{k}\right)$$

Here, *h^k^* denotes the output feature maps, *b^k^* refers to the bias, *W^k^* refer to the weights, and *x* is the input image (56). For input *X* in a convolutional layer, the process can be mathematically represented as (57):


(9) $$\text{con}=f\left(\sum_{i,j\in M}X_{ij}=W_{m-i,n-j}+b\right)$$

where *M* is the convolutional area, *x* is the element in area *M, w* denotes the element of the convolutional kernel, *m, n* is the size of the kernel, *b* refers to the offset, and *f* (.) refers to the activation function of the convolutional layer. For the pooling layer process, the mathematical expression is (57):


(10) $$\text{pool}=\text{down}\left(max\left(y_{i,j}\right)\right),i,j\in p$$

where *p* represents the pool area, *y* denotes the element in the area *p*, and down () refers to the down sampling method, which preserves the maximum value from *p*.

#### Ablation study

In order to determine the optimal layer architecture and configuration of a CNN model, the nature and characteristics of a task and possible related challenges should be considered (58). The aim of the ablation study is to acquire a clear understanding of the model’s performance by analyzing the consequence of altering some components (59). With the alteration of different components or hyper-parameters of a model, a change in performance is observed (60). This method can ascertain any potential decrease in the performance of the model, which can later be fixed by updating and tuning the network. Therefore, we have trained our base CNN model several times by altering layer numbers, filer sizes, filer numbers, hyper-parameters, and parameter values to obtain an optimal performance with low computational complexity. All the experiments are performed on the breast cancer mammogram dataset as this is a challenging dataset that contains artifacts, noise, a limited number of images, similarities between intra-classes, and intensity similarity between suspicious regions and surrounding healthy tissues. If a model can address all these challenges, it can be assumed that it might also provide good outcomes for the rest of the datasets. The results of the ablation study can be found in Section “Results of the ablation study.”

#### Dataset split and training strategy

All the datasets are split using a 70:20:10 ratio for training, validation, and testing, respectively, of the datasets. “Categorical cross-entropy,” specified as “categorical_cross entropy” (61) is a multi-class cross entropy found in Keras and is utilized as the loss function while compiling the model. The cross-entropy loss function is typically applied to a feature discrimination network. The relevant equations are as follows (62):


(11) $$\text{Loss}\left(d,v\right)=-\sum_{j=0}^{m}\sum_{i=0}^{n}\left(d_{ij}*\text{log}\left({\hat{v}}_{ij}\right)\right)$$

where *d* represents true label and predicted label is represented with *v*. The batch size of the dataset is denoted by *m*, with n being the number of classes. ${\hat{v}}_{ij}$ is the probability predicted by the model at ith observation on jth category. Since the training of neural networks is computationally intensive, especially with a large dataset, it is crucial to utilize graphical processing units (GPUs). Three computers equipped with Intel Core i5-8400 Processor, NVidia GeForce GTX 1660 GPU, 16 GB of memory, and 256 GB DDR4 SSD for storage are used for this research.

#### MNet-10

As deep networks tend to consume more computational resources and time, the approach of employing shallow architecture is applied to address time and computational complexity. Our proposed architecture contains several modules and layers, including the input layer, convolutional layers, activation function, pooling layers, a fully connected layer, dropout, and an output dense layer.

**FIGURE 6:**
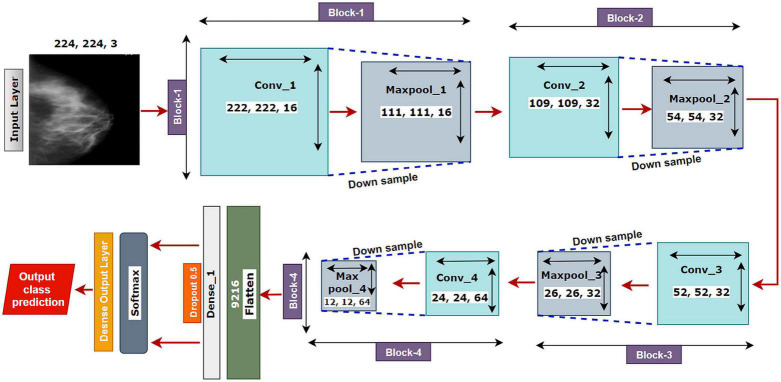
The architecture of the proposed MNet-10 model after ablation study.

The proposed model MNet-10 (Figure 6) contains a total of 10 layers including four convolutional layers, four max-pooling layers, and two dense layers. The ten layers are determined after carrying out extensive experiments on the dataset performing an ablation study. Among them, the four convolutional layers and the last two dense layers are considered as weighted layers. A flatten layer is introduced before the dense layers. A total of four blocks are present in this architecture where each block contains a 3 × 3 kernel-sized convolutional layers followed by a max-pooling layer of kernel size 2 × 2. All the convolutional layers are equipped with the PReLU non-linear activation function and have a stride size of 1 × 1. The filters or kernels in the 2D convolutional layers are made up of a set of weights that determines what features to detect from the input image (63). The weights can be considered as parameters that get updated after every epoch (64). The first two convolutional layers are used to extract textural features (edges and corners) from the input image while the other layers are used for a more abstract representation of the input data containing complex shapes and deep textural features (65). MNet-10 has a total of 10,768,292 trainable parameters. While training the model, the initial weights extract features from the input data, and the error rate of the network is calculated through the loss function. Afterward, after every training epoch, the weights of all the kernels are modified based on error rate. This way, the kernels are altered after every epoch and optimal features can be extracted.

The input layer is fed to Block-1 where the first convolutional layer has 16 filters containing a total of 788,992 trainable parameters that extract trivial features from the input RGB images. As the first layer works with the input images, in extracting only relevant patterns such as edges and corners from mammograms, it is important to extract only relevant data in this layer to lessen the number of unwanted features for other convolutional layers to help with better generalization of the ROI region, and lower number of feature maps lessens computational complexity. In this regard, the Block-1 convolutional layer is comprised of a low number of 16 filters that maintain structural details while keeping distinguishing textural characteristics of the input mammograms. This produces a total of 16 feature maps for every input data, which afterward get rectified with PReLU keeping only the non-negative values of the feature maps. Afterward, a 2 × 2 max-pool layer scales down the resulted feature maps from the first convolutional layer into half its size. This layer picks the highest pixel values from every 2- × -2 area of the 222- × -222-sized rectified feature maps and constructs smaller 111- × -111-pixel-sized pooled feature maps with the highest pixel values. The max-pool layers of the following Blocks have the same working principle. As the dimensions of the feature maps are reduced, the following convolutional layer has much smaller data to analyze, which in turn plays a big role in lessening computational complexity. The pooled feature maps are passed as input data for Block-2.

Block-2 and Block-3 comprise a 3- × -3-kernel-sized convolutional layer of 32 filters with 384,832 and 95,776 trainable parameters, respectively, and are equipped with a PReLU activation function. Moreover, each convolutional layer of Block-2 and Block-3 is followed by a max-pool layer of 2 × 2. Block-2 and Block-3 extract more features from the feature maps produced by Block-1 and scale down the resulting feature maps to half their size. A CNN network’s ability to extract more abstractions from visual inputs increases with the number of filters of convolutional layers. In this regard, the convolutional layer filter number is increased to 32 filters for Block-2 and Block-3 to extract more distinct textural feature maps. The increase in filter size is subtle (32 filters) keeping time complexity and ROI generalization capabilities in mind. Just like the functionalities of Block-1, a convolutional layer of Block-2 extracts a total of 32 feature maps of 109 pixels × 109 pixels that get rectified by PReLU and pooled by a max-pool layer resulting in 32 feature maps of 54 pixels × 54 pixels. The feature maps are later passed to Block-3 that produces additional 32 feature maps of 26 pixels × 26 pixels. The resulting feature maps of this Block contain a more abstract representation of the input data containing various shapes and objects of the images that are complex. The feature maps are used as input for Block-4.

Block-4 includes the 3- × -3-kernel-sized convolutional layer with 64 filters with a total of 55,360 trainable parameters and a max-pooling layer with a 2- × -2-sized kernel. PReLU is equipped in this layer to produce rectified feature maps as seen in the previous Blocks. In order to extract a greater number of abstractions from the input data, the filter size of this layer is increased to 64, which is considered as a subsequent amount of feature maps for generalizing the input data while maintaining lower computational complexity. The feature maps contain more deep features of input data. The resulting feature maps of the convolutional layer of Block-4 have a dimension of 24 × 24. Afterward, the max-pool layer scales down the feature maps to 12 pixels × 12 pixels, hence reducing computational complexity while conserving important features of the input image. A total of 64 feature maps are produced by Block-4 that contains additional deep features of the input data with more complex shapes and objects than the previous Blocks.

The resultant multidimensional feature maps of Block-4 are flattened into a 1D vector containing 9,216 values for each mammogram. The flatten layer is followed by a fully connected (FC) layer that contains 1,024 neurons equipped with the PreLU activation function. Each value of the resulting 1D array serves as input neuron for the first FC layer where each input neuron is connected to each neuron present in the first FC layer. This connection of input neurons to FC neurons is called weights that can be updated after each epoch by backpropagation. Weights are responsible for generalizing the extracted features of convolutional layers by associating features to a particular class. The first FC layer is followed by a dropout layer with a value of 0.5. Afterward, a second FC layer, which is considered a classification layer containing four neurons and equipped with a softmax activation function (66), is utilized for classifying the input mammograms into four classes. Each resulting neuron of the dropout layer is connected to each neuron of the second FC layer. This layer further generalizes the features, and the softmax activation function gives prediction scores for all four classes (BC, BM, MC, and MM). The error rate is calculated through the categorical loss function, and the weights of the fully connected layer and convolutional layers are updated after every epoch depending on the error rate.


(12) $$softmax\left(z_{i}\right)=\frac{e^{z_{i}}}{\Sigma_{j}e^{z_{j}}}$$

The mathematical expression of the Softmax function is described in equation (28), where *z_i_* refers to the outputs of the output neurons and inputs of the Softmax function. Exp () is a non-linear exponential function that is applied to each value of*z_i_*. The bottom part of equation (28) (Σ_*j*_*exp*(*z*_*i*_)) normalizes the exponential values (*exp*(*z*_*i*_)) by dividing them with the summation of *exp*(*z*_*i*_).

## Results and discussion

This section solely focuses on the presentation and discussion of the results and key findings of this research. This includes the results of the ablation study and performance analysis of the proposed model on multiple medical image datasets. Furthermore, comparisons of various data augmentation techniques and their impact on a particular medical image dataset are also discussed in this section.

### Evaluation matrices

To evaluate the performance of all the experiments including the ablation study, different augmentation techniques and three deep learning models, several evaluation metrics, namely, precision, recall, F1-score, accuracy (ACC), sensitivity, the area under the curve (AUC), and specificity, are used. A confusion matrix is generated for each experiment from which the values of true positive (TP), true negative (TN), false positive (FP), and false negative (FN) cases are derived. AUC value is the resultant of the receiver operating characteristic (ROC) curve that plots the true positive rate (TPR) against the false positive rate (FPR) at various threshold values. TPR is an alternative term for Recall. The necessary formula can be stated as follows (65, 67):


(13) $$\text{ACC}=\frac{\text{TP}+\text{TN}}{\text{TP}+\text{TN}+\text{FP}+\text{FN}}$$

(14) $$\text{Recall}=\frac{\text{TP}}{\text{TP}+\text{FN}}$$

(15) $$\text{Specificity}=\frac{\text{TN}}{\text{TN}+\text{FP}}$$

(16) $$\text{Precision}=\frac{\text{TP}}{\text{TP}+\text{FP}}$$

(17) $${\text{F}}_{1}=2\frac{\mathrm{precision}*\mathrm{recall}}{\text{precision}+\text{recall}}$$

### Results of the ablation study

All components of the base CNN architecture are altered, and the results are recorded. For each case study, we show the time complexity (68), training time per epoch, and test accuracy. Theoretical time complexity can be defined as (69):


(18) $$O=\left\{\sum_{j=1}^{k}n_{j-1}\cdot s_{w}\cdot s_{h}\cdot n_{j}\cdot m_{w}\cdot m_{h}\right\}$$

where *j* refers to the index number of each convolutional layer and *k* denotes the total number of convolutional layers, *n*_*j–1*_ is the total number of the kernel or input channels in the *j* – 1st convolutional layer, *n_j_* denotes the total number of kernels or output channels in the jth layer, *s_w_* and *s_h_* denote the width and height of the kernels individually, and *m_w_* and *m_h_* refer to the width and height, respectively, of the generated feature map.

The results of the entire ablation study are presented in Tables 3, 4. Table 3 contains all the results related to the model’s layer configurations and activation functions, and Table 4 presents the results of tuning hyper-parameters, the loss function, and flatten layer.

**TABLE 3:** Ablation study on layer configurations and activation functions.

Case study 1: changing convolution and maxpool layer							
Configuration no.	No. of convolution layer	No. of pooling layer	Time complexity	Epoch × training time		Test accuracy (%)	Finding
1	5	5	66M	79 × 54s		89.55	Modest accuracy
2	4	4	64M	75 × 54s		93.36	Highest accuracy
3	3	3	62M	79 × 54s		86.27	Lowest accuracy
4	6	6	64M	84 × 56s		91.15	Modest accuracy
5	7	7	–	–		–	Error

**Case study 2: changing filter size**							

**Configuration no.**	**Filter size**	**Time complexity**	**Epoch × training time**		**Test accuracy (%)**		**Finding**

1	3 × 3	64M	72 × 54s		93.36		Near highest accuracy
2	2 × 2	28M	78 × 55s		93.07		Highest accuracy
3	5 × 5	178M	82 × 55s		93.47		Highest accuracy

**Case study 3: changing the number of filter**							

**Configuration no.**	**No. of kernel**	**Time complexity**	**Epoch × training time**		**Test accuracy (%)**		**Finding**

1	64 → 64 → 64 → 64	28M	75 × 54s		93.36		Modest accuracy
2	32 → 32 → 32 → 32	14M	83 × 53s		91.22		Accuracy dropped
3	32 → 32 → 64 → 64	16M	79 × 53s		94.51		Accuracy improved
4	16 → 32 → 32 → 64	10M	71 × 51s		94.75		Highest accuracy

**Case study 4: changing type of pooling layer**							

**Configuration no.**	**Type of pooling layer**	**Time complexity**	**Epoch × training time**		**Test accuracy (%)**		**Finding**

**1**	Max	10M	66 × 51s		94.75		Highest accuracy
2	Average	10M	71 × 52s		94.75		Highest accuracy

**Case study 5: changing activation function**							

**Configuration no.**	**Activation function**	**No. of parameter**	**Epoch × training time**		**Test accuracy (%)**		**Finding**

1	PReLU	10M	71 × 55s		96.52		Highest accuracy
2	Relu	10M	66 × 51s		94.75		Previous accuracy
3	Leaky ReLu	10M	78 × 59s		95.66		Accuracy improved
4	Tanh	10M	78 × 60s		94.2		Accuracy dropped
5	ELU	10M	78 × 57s		96.17		Accuracy improved

**TABLE 4:** Ablation study on model hyper-parameters, loss function, and flatten layer.

Case study 6: changing batch size					
Configuration no.	Batch size	Time complexity	Epoch × training time	Test accuracy (%)	Finding
1	16	10M	71 × 59s	95.84	Accuracy dropped
2	32	10M	68 × 56s	96.83	Highest accuracy
3	64	10M	71 × 55s	96.52	Previous accuracy
4	128	10M	78 × 51s	96.28	Accuracy dropped

**Case study 7: changing flatten layer**					

**Configuration no.**	**Flatten layer type**	**Time complexity**	**Epoch × training time**	**Test accuracy (%)**	**Finding**

1	Flatten	10M	68 × 56s	96.83	Highest accuracy
2	Global max pooling	10M	75 × 56s	96.47	Accuracy dropped
3	Global average pooling	10M	83 × 58s	96.38	Accuracy dropped

**Case study 8: changing loss functions**					

**Configuration no.**	**Loss function**	**Time complexity**	**Epoch × training time**	**Test accuracy (%)**	**Finding**

1	Binary crossentropy	10M	82 × 56s	88.57	Accuracy dropped
2	Categorical crossentropy	10M	68 × 56s	96.83	Highest accuracy
3	Mean squared error	10M	73 × 55s	87.62	Accuracy dropped
4	Mean absolute error	10M	92 × 56s	74.80	Accuracy dropped
5	Mean squared logarithmic error	10M	68 × 56s	95.81	Accuracy dropped
6	Kullback Leibler divergence	10M	78 × 56s	96.04	Accuracy dropped

**Case study 9: changing optimizer**					

**Configuration no.**	**Optimizer**	**Time complexity**	**Epoch × training time**	**Test accuracy (%)**	**Finding**

1	Adam	10M	68 × 56s	96.83	Accuracy dropped
2	Nadam	10M	74 × 56s	97.15	Highest accuracy
3	SGD	10M	87 × 61s	92.68	Accuracy dropped
4	Adamax	10M	89 × 58s	95.75	Accuracy dropped
5	RMSprop	10M	91 × 59s	90.82	Accuracy dropped

**Case study 10: changing learning rate**					

**Configuration no.**	**Learning rate**	**Time complexity**	**Epoch × training time**	**Test accuracy (%)**	**Finding**

1	0.01	10M	92 × 55s	91.46	Accuracy dropped
2	0.007	10M	87 × 56s	95.85	Accuracy dropped
3	0.001	10M	74 × 56s	97.15	Previous accuracy
4	0.0007	10M	65 × 57s	97.34	Highest accuracy
5	0.0001	10M	68 × 57s	97.28	Accuracy improved

#### Case study 1: Changing convolutional and max-pool layers

In this case study, the configuration mentioned above is kept as it is, while the number of convolutional and max-pool layers is changed. Initially, we start with five convolution layers followed by five max-pool layers. Table 3 shows the performance of different configurations of the model architecture with the total number of parameters and training time. The best performance is achieved by configuration 2 (Table 3) with an accuracy of 93.36%. We get the highest accuracy for this configuration within 75 epochs, and the training time per epoch was 54 s, which is the lowest training time. Configuration 2 consists of four pairs of convolutional and max-pool layers. This configuration was selected for the rest of the ablation case studies.

#### Case study 2: Changing filter size

In this case study, we have experimented with different kernel sizes of 3 × 3, 2 × 2, and 5 × 5 to observe performance (70). It is observed that changing filter size does not much affect the overall performance (Table 3). However, the highest accuracy, 93.47%, is achieved when employing the kernel size 5 × 5 with the training time per epoch requiring 55 s. Filter 3 × 3 had the second highest accuracy of 93.36% with an epoch time of 54 s. Filter size 3 × 3 reached its top accuracy in 72 epochs and the 5 × 5 kernel in 82 epochs where 3 × 3 had a lower per epoch training time of 54 s. Filter size 3 × 3 had a lower time complexity (64 million) than filter size 5 × 5 (178 million). As filter size 3 × 3 recorded nearly the highest accuracy while maintaining low time complexity as well as low epoch numbers and training time, this configuration is chosen for further ablation case studies.

#### Case study 3: Changing the number of filters

Initially, we started with a constant number of kernels (58) for all the four convolutional layers (64 → 64 → 64 → 64). Later, the number of features is reduced to 32, and no improvement in performance is found. However, we anticipated that gradually increasing might be a better approach. This is represented in configurations 3 and 4 (Table 3). It is evident that configuration 4 with filter numbers 16, 32, 32, and 64 for the four convolutional layers achieved the highest performance with a test accuracy of 94.75% and the lowest time complexity and model training time. Therefore, we move forward with configuration 4.

#### Case study 4: Changing the type of pooling layer

Two pooling layers, max pool and average pool, are evaluated (68), with both pooling layers gaining the same highest accuracy of 94.75% (Table 3). It is observed that the max pooling layer required a lower epoch number of 66 to achieve the highest accuracy while maintaining a low training time per epoch of 51 s. The max pooling layer is therefore chosen for further ablation studies.

#### Case study 5: Changing the activation function

As different activation functions can impact the performance of a neural network model, choosing an optimal activation function is gaining a relevant research question. Five activation functions, PReLU, ReLU, Leaky ReLU, Tanh, and Exponential Linear Units (ELUs) (71) are experimented with. PRelu performs best with a test accuracy of 96.52% (Table 3). This activation function was chosen for further ablation studies.

#### Case study 6: Changing batch size

Batch size denotes the number of images used during each epoch to train the model. A larger batch size may result in the model taking a long time to accomplish convergence while a smaller batch size can cause poor performance. Moreover, performance varies for different batch sizes of medical datasets because of the complex structure of medical images (29). We have experimented with four batch sizes and found that both the batch sizes of 16 and 32 achieved the highest accuracy of 96.8% (Table 4). However, the batch size of 32 results in better overall performance, maintaining lower epoch numbers and training times than the batch size of 16. Therefore, a batch size of 32 is chosen for further ablation studies.

#### Case study 7: Changing flatten layer

A flatten layer takes the multidimensional output of previous layers and produces a one-dimensional tensor. We experimented with Global Max pooling and Global Average pooling instead and found that the previously used flatten layer yielded the highest test accuracy of 96.83% (Table 4) while maintaining the lowest training time.

#### Case study 8: Changing loss functions

Experimentation with different loss functions including Binary Crossentropy, Categorical Crossentropy, Mean Squared Error, Mean Absolute Error, Mean Squared Logarithmic Error, and Kullback Leibler Divergence was carried out to select the appropriate loss function for our network. While equipped with Categorical Crossentropy, the model had a 96.83% (Table 4) test accuracy, which is the best result. Hence this is chosen.

#### Case study 9: Changing optimizer

Experimentation with different optimizers including Adam, Nadam, SGD, Adamax, and RMSprop was carried out to identify the optimal optimizer. In this case, the learning rate was set to 0.001. The best test accuracy of 97.15% (Table 4) was recorded with the Nadam optimizer. We select the Nadam optimizer for further ablation study.

#### Case study 10: Changing learning rate

An experimentation with different learning rates of 0.01, 0.005, 0.001, 0.0005, and 0.0001 was conducted. The best test accuracy of 97.34% (Table 4) was recorded with a learning rate of 0.0005 and the Nadam optimizer.

**FIGURE 7:**
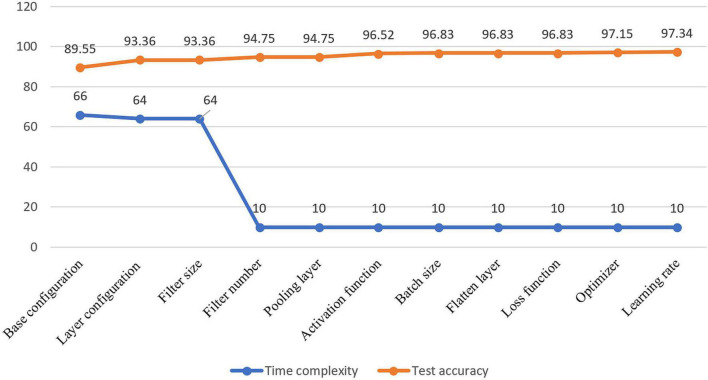
Visualization of resulting time complexity (measured in millions and scaled into range 0–100) and test accuracy (measured in percentage) of all the ablation case studies.

Visual representation of gradual performance boost with different ablation study cases and gradual decrease in time complexity is shown in Figure 7 for better understanding.

### Results of different datasets for different augmentation techniques

After the ablation study, the optimal model configuration is used for further analysis. The model is trained with each of the datasets described before and after conducting augmentation. As mentioned, two augmentation approaches were performed for each dataset, leading to three sets of results, including the result of the dataset before augmentation as shown in Table 5. Afterward, to further assess our model’s robustness, three deep learning algorithms, VGG16, InceptionV3, and ResNet50, are trained on each of the datasets. In this regard, the augmented dataset for which the best performance is achieved is used to train the deep learning models. In this section, the results are explained along with a discussion at the end of the section.

**TABLE 5:** Results of datasets breast mammogram, skin cancer, chest X-ray, tympanic membrane, brain tumor MRI, chest cancer CT-scan, breast cancer microscopic biopsy image, and breast cancer ultrasound image.

(1) Breast mammogram dataset											
Experiment	T_acc	T_loss	Val_acc	V_loss	Te_acc	Te_loss	Precision	Recall	Specificity	F1_score	AUC
Before augmentation	76.13	0.48	69.07	0.49	66.84	0.46	66.76	66.83	79.13	70.26	66.95
Geometric	95.35	0.12	94.08	0.23	90.32	0.24	90.79	90.39	96.37	90.59	90.43
**Photometric**	**93.90**	**0.16**	**96.91**	**0.08**	**97.34**	**0.08**	**97.34**	**97.10**	**98.95**	**97.12**	**97.47**

**(2) Skin cancer dermoscopy dataset**											

**Experiment**	**T_acc**	**T_loss**	**Val_acc**	**V_loss**	**Te_acc**	**Te_loss**	**Precision**	**Recall**	**Specificity**	**F1_score**	**AUC**

Before augmentation	90.13	0.28	89.37	0.54	88.86	0.28	88.34	88.64	93.25	88.49	88.92
Geometric	97.68	0.18	97.40	0.48	97.82	0.1872	97.71	97.76	99.06	97.73	97.91
**Photometric**	**96.43**	**0.016**	**94.04**	**0.02**	**98.43**	**0.016**	**98.24**	**98.56**	**99.32**	**98.40**	**98.65**

**(3) COVID chest X-ray dataset**											

**Experiment**	**T_acc**	**T_loss**	**Val_acc**	**V_loss**	**Te_acc**	**Te_loss**	**Precision**	**Recall**	**Specificity**	**F1_score**	**AUC**

Before augmentation	76.15	0.10	75.02	0.32	73.66	0.10	73.36	73.65	84.56	73.47	73.77
Geometric	98.39	0.09	98.16	0.23	94.81	0.09	94.53	94.54	97.05	95.53	94.95
**Photometric**	**94.86**	**0.15**	**97.48**	**0.07**	**97.29**	**0.07**	**97.32**	**97.31**	**99.09**	**97.31**	**97.42**

**(4) Tympanic membrane dataset**											

**Experiment**	**T_acc**	**T_loss**	**Val_acc**	**V_loss**	**Te_acc**	**Te_loss**	**Precision**	**Recall**	**Specificity**	**F1_score**	**AUC**

Before augmentation	65.15	0.82	64.52	0.08	64.37	0.08	63.82	64.06	75.48	63.94	64.41
Geometric	98.02	0.07	88.85	0.54	92.10	0.04	86.99	89.10	96.55	88.04	92.23
**Photometric**	**97.50**	**0.08**	**96.81**	**0.15**	**96.31**	**0.12**	**96.28**	**96.40**	**98.74**	**96.34**	**96.48**

**(5) Brain tumor MRI dataset**											

**Experiment**	**T_acc**	**T_loss**	**Val_acc**	**V_loss**	**Te_acc**	**Te_loss**	**Precision**	**Recall**	**Specificity**	**F1_score**	**AUC**

Before augmentation	90.13	0.28	84.07	0.49	82.36	0.366	82.27	82.56	89.13	82.41	82.44
Geometric	98.18	0.06	98.62	0.06	98.93	0.05	98.93	99.0	99.63	98.97	99.04
**Photometric**	**98.82**	**0.04**	**99.42**	**0.03**	**99.54**	**0.04**	**99.54**	**99.59**	**99.84**	**99.56**	**99.71**

**(6) Chest cancer CT-scan dataset**											

**Experiment**	**T_acc**	**T_loss**	**Val_acc**	**V_loss**	**Te_acc**	**Te_loss**	**Precision**	**Recall**	**Specificity**	**F1_score**	**AUC**

Before augmentation	65.15	0.82	64.52	0.08	64.37	0.08	63.82	64.26	81.45	64.03	64.41
Geometric	98.78	0.03	97.75	0.10	97.56	0.10	97.63	97.63	99.16	97.63	97.84
**Photometric**	**98.89**	**0.03**	**99.59**	**0.04**	**99.82**	**0.31**	**99.82**	**99.85**	**99.91**	**99.84**	**99.90**

**(7) Breast cancer microscopic biopsy image dataset**											

**Experiment**	**T_acc**	**T_loss**	**Val_acc**	**V_loss**	**Te_acc**	**Te_loss**	**Precision**	**Recall**	**Specificity**	**F1_score**	**AUC**

Before augmentation	91.15	0.40	85.02	0.42	83.66	0.10	83.36	83.65	84.56	83.65	83.80
**Geometric**	**97.93**	**0.06**	**97.63**	**0.08**	**96.76**	**0.06**	**96.40**	**96.18**	**98.53**	**96.29**	**96.84**
Photometric	95.06	0.06	93.87	0.22	93.50	0.15	92.06	93.08	95.86	92.57	93.63

**(8) Breast cancer ultrasound image dataset**											

**Experiment**	**T_acc**	**T_loss**	**Val_acc**	**V_loss**	**Te_acc**	**Te_loss**	**Precision**	**Recall**	**Specificity**	**F1_score**	**AUC**

Before augmentation	76.15	0.10	75.02	0.32	73.66	0.10	73.36	73.65	84.56	73.47	73.81
Geometric	98.39	0.12	98.16	0.23	95.38	0.09	95.53	95.54	97.05	95.53	95.55
P**hotometric**	98.95	**0.06**	**98.63**	**0.05**	**97.45**	**0.06**	**97.49**	**97.72**	**99.18**	**97.61**	**97.59**

The results include training accuracy (T_acc), training loss (T_loss), validation accuracy (V_acc), validation loss (V_loss), test accuracy (Te_acc), test loss (Te_loss), precision, recall, specificity, F1 score, and area under the curve value (AUC).

Table 5 presents the computed results of our proposed model, MNet-10, evaluated on all the eight medical image datasets, both augmented and non-augmented. While testing the proposed model on the Breast mammogram, Breast cancer ultrasound, and tympanic membrane datasets, the highest test accuracies of 97.34, 98.75, and 96.31% were achieved utilizing the photometric augmentation technique. Similarly, testing the proposed model on the skin cancer dermoscopy, COVID chest X- dataset, chest CT scan, and Brain tumor MRI datasets, the findings indicate that the photometric augmentation technique prevails with accuracies of 98.43, 97.29, 99.82, and 99.54%, respectively. On the other hand, the geometric augmentation technique recorded a higher accuracy in terms of the breast cancer microscopic biopsy image dataset with an accuracy of 96.76%.

**FIGURE 8:**
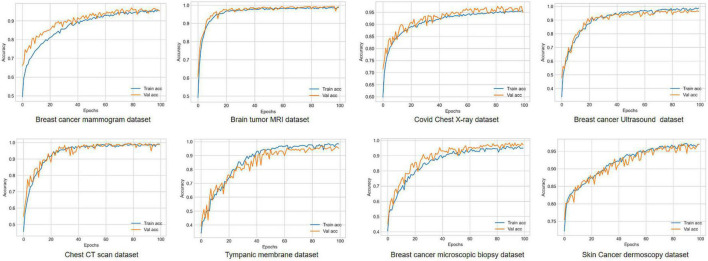
Accuracy curves for all the eight medical image datasets trained on proposed MNet-10.

Figure 8 shows the accuracy curves for proposed MNet-10 on the best performing augmented datasets of all the medical image datasets. It is observed from all the eight accuracy curves that the training curve converges smoothly from the first to the last epoch showing approximately no bumps. The difference between the training accuracy and validation accuracy curve is minimal. In conclusion, after analyzing the training, no evidence of overfitting is found.

### A brief discussion of the augmentation results

Regarding image augmentation techniques, various outcomes can be observed from the eight different modalities. In most cases, the photometric image augmentation technique yields a better outcome in terms of test accuracies than the geometric augmentation technique (Table 5). For mammogram images, photometric augmentation provided the highest test accuracy of 97.34% (Table 5), whereas the accuracy is drastically reduced for geometric augmentation (90.32%). We assume that the reason behind this is the changing of the position of the cancer region (ROI) in the mammograms with the geometric augmentation technique. Breast cancer mammograms contain cancerous ROIs that are often hard to distinguish from dense tissues as they appear bright. Hence, because of the nature of the datasets, to successfully classify mammograms, it is important to preserve the ROI structure and position of the images as much as possible while augmenting them. With geometric augmentation techniques for complex datasets, the ROIs of resultant augmented images change position to an extent that less resembles real-world mammograms. Moreover, with the geometric augmentation technique, the position and details of ROIs in a complex medical image can be heavily altered, resulting in the loss of ROI information. It can be said that, along with other features, the geometric position is a crucial feature for these datasets and impacts greatly the performance of a model. While training a model with such augmented images, some features learned by the model may not even be related to features of a real-word test dataset. As a consequence, when a geometric axis is altered, the model tends to obtain results with higher false negative rates when differentiating classes.

Therefore, for images where the ROI is complex, hidden and geometrical information is important; applying geometric augmentation might not be a good approach. On the other hand, with the photometric augmentation technique, the position of the cancer region is not affected; rather, the intensity of the ROI changes, resulting in augmented images that are not highly dissimilar to the original images. With this approach, as the geometric perspective is not altered, the structural information of the ROI is preserved quite accurately so the resulting image is close to real-world datasets. Deep learning models trained with augmented images show better performance in terms of prediction rates on test datasets. This improves the model’s understanding of cancer regions and their positions and gives better predictions on test datasets.

In our study, the photometric augmentation technique provided the highest performance for the Chest CT scan, COVID chest X-ray, Tympanic membrane, and breast cancer ultrasound image datasets, with test accuracies of 99.82, 97.29, 96.31, and 98.75%, respectively. With the geometric augmented technique, the datasets showed a 3–5% decrease in test accuracies. The ROIs contained in the four datasets are less complex, and the ROI region is more defined than the surrounding regions. Hence, the differences between classes are more easily distinguishable than mammograms. For these datasets, a 2–5% accuracy drop is observed while training with the geometric augmentation technique over photometric augmentation technique, whereas for a complex dataset like that of a mammogram, the accuracy fell drastically (>7%).

We obtained near-identical accuracies, with an accuracy difference of around 1% between both of the augmentation techniques on the Skin cancer dermoscopy and Brain tumor MRI datasets. In the skin cancer dermoscopy images, the achieved test accuracies are 98.43 and 97.82%; also, in the Brain tumor MRI images, 99.54 and 98.93% were achieved, respectively, for the photometric and geometric augmentation techniques. It is found that the ROIs of the datasets are quite straightforward, clearly visible, and less complex. In this case, the geometric augmentation technique does not much affect the structural information of the ROI to a large extent. For this reason, the types of datasets can be expected to perform quite well with both augmentation techniques as geometric alteration has less effect on distinguishing the ROI.

In terms of the breast cancer microscopic image dataset, the geometric augmentation technique acquired a test accuracy of 96.76%, which is about 3% higher than that of the photometric augmentation technique (93.5%). We assume that the reason behind the success of the geometric technique in this regard lies in the characteristics of the images in this dataset. The histopathological images of the breast cancer microscopic dataset are in a very high-quality RGB format where the contrast and brightness levels of the images are well-adjusted. The ROI regions in histopathological images are very straightforward and very distinguishable from the background pixels where geometric alteration does not change any necessary information. While applying the photometric augmentation technique to such images, the ROI regions might get overexposed with an increase in brightness or get underexposed with a reduction in brightness. Overexposed and underexposed images can result in the loss of ROI information in a histopathological image. On the other hand, with the geometric augmentation technique, pixel intensity is not affected. Consequently, for a high-quality image dataset such as the breast cancer microscopic image dataset, the geometric augmentation technique can perform a bit better. In general, the photometric augmentation technique clearly seems to achieve better performance, but the geometric augmentation technique performs moderately in some cases while in other cases a drastic decrease in performance is observed. As various medical datasets contain different characteristics, rigorous observations with multiple augmentation techniques should be carried out to find the best-performing augmentation technique for a particular dataset.

Alongside the photometric and geometric augmentation methods, the elastic deformation data augmentation technique is also utilized to observe how it performs on the eight datasets. This is considered one of the most complex kinds of augmentation, as it can heavily alter an image. It is quite similar to stretching an image, but overdoing elastic deformation can lead to distorted training images.

In elastic deformation, deformation intensity is denoted with sigma (σ). With sigma values higher than 20, the resulting augmented images become quite distorted. Hence, all the eight datasets are augmented four times with four different σ values of 5, 10, 15, and 20. Afterward, the proposed model is tested again with the augmented datasets, and the results are recorded. For the datasets of Breast mammogram, COVID chest X-ray, and chest cancer CT scan, the obtained test accuracies of MNet-10 are 84.45, 87.31, and 91.55%, respectively, with the elastic deformation augmentation technique. This performance is quite lower than the accuracies obtained from both the photometric and geometric augmentation techniques, while the highest accuracy in the range of 97%-99% was gained with the photometric augmentation technique. On the contrary, the Skin cancer dermoscopy, Tympanic membrane, breast cancer microscopic biopsy image, breast cancer ultrasound image, and Brain tumor MRI datasets augmented with the elastic deformation technique showed accuracies of 97.41, 91.85, 95.83, 94.96, and 94.17%, which are quite close to the accuracies obtained with the geometric augmented datasets. In this regard, the traditional photometric and geometric augmentation techniques seemed to outperform the elastic augmentation technique in most cases.

### Performance comparison with state-of-the-art deep learning models

In this section, the proposed MNet-10 model is further evaluated by comparing it with some state-of-the-art transfer learning models, namely, VGG16, ResNet50, and Inception V3, on the best performing augmented datasets. In this regard, we have chosen the three models based on various research studies conducted on similar medical datasets in recent times. The growth of smart medicine is strongly supported by various CNN models such as VGG16 and ResNet (72), which are considered the most popular transfer learning models for analyzing medical images (73). Furthermore, these models can be used in datasets similar to ours (40, 41, 72, 74, 75). Also, InceptionV3 has been used on datasets (76–78) similar to ours. Although these models are a bit old, they are well-established and have been proven to be quite effective in numerous research studies. As these models represent three very different types of CNN architectures offering different numbers of parameters (ranging from 23 to 143 million), they tend to perform differently with various small and big medical datasets. Being three very different types of state-of-the-art models, they can give an insight into their raw performance on the eight medical datasets and pose a fair performance comparison with the MNet-10 model. For these reasons, VGG16, ResNet50, and InceptionV3 have been chosen for comparison.

These models are trained for 100 epochs using the optimizer Nadam, a learning rate of 0.0007, and a batch size of 32 as this is the optimal hyper-parameter configuration for our proposed MNet-10 model. The results of this comparison are presented in Table 6. Across all the medical datasets, our proposed Mnet-10 is found to outperform the other three models in the comparison. A common observation for the VGG16, Inception V3, and ResNet50 models is that for some datasets, the performance is quite satisfactory while for others the performance is noticeably reduced. However, the VGG16 model performed better than the ResNet50 and InceptionV3 models on datasets that contain small and quite complex ROIs such as the mammogram image, COVID chest X-ray, brain tumor MRI, and chest CT scan datasets (Table 6). On the other hand, the InceptionV3 model outperformed VGG16 in terms of datasets containing big and obvious ROIs such as skin cancer, tympanic membrane, and breast cancer microscopic biopsy datasets. ResNet50 performed noticeably poorly in the comparison, with the majority of the accuracies dropping below 80% (Table 6). Furthermore, various statistical measures (79) besides test accuracy are also calculated for all the models including F1 score, specificity, and AUC values (Table 6) where MNet-10 seems to outperform all the models. Unlike test accuracy, the three CNN models (VGG16, Inception V3, and ResNet50) were unable to produce consistent performance across all datasets in terms of F1 score, specificity, and AUC. ResNet50 also seemed to fall behind both VGG16 and InceptionV3 in this regard. This further adds to the robustness of the proposed model. Moreover, a Wilcoxon signed-rank test (80) is also conducted to highlight the statistical significance between the results produced by the proposed network and the other models shown in Table 6. In this regard, a *P*-value of less than 0.05 is considered a significant level (81). Table 7 showcases the findings of the Wilcoxon signed-rank test conducted with F1 scores (Table 6). The outcome of this test shows an achieved *P*-value of 0.003 in all the cases (Table 7) and concludes that the performance difference between the proposed MNet-10 and the other DL models is quite statistically significant.

**TABLE 6:** Results of VGG16, ResNet50, InceptionV3, and MNet-10 on breast mammogram, skin cancer, chest X-ray, tympanic membrane, brain tumor MRI, chest cancer CT scan, and breast cancer ultrasound image with photometric augmentation techniques and breast cancer microscopic biopsy image with geometric augmentation techniques.

Datasets	Statistical tests	VGG16	ResNet50	InceptionV3	Proposed model
Breast Mammogram dataset	Test accuracy	90.10	63.82	88.24	97.34
	F1 score	89.47	59.92	88.15	97.12
	AUC	91.38	63.97	89.32	97.47
	Specificity	93.61	68.45	93.49	98.95
Skin cancer dermoscopy dataset	Test accuracy	90.68	82.71	92.19	98.43
	F1 score	87.18	81.35	90.26	98.40
	AUC	92.04	82.11	93.84	98.65
	Specificity	94.12	86.09	96.17	99.32
COVID chest X-ray dataset	Test accuracy	93.74	78.80	89.87	97.29
	F1 score	92.36	75.63	86.95	97.31
	AUC	95.27	76.29	90.32	97.42
	Specificity	95.41	83.93	92.40	99.09
Tympanic membrane dataset	Test accuracy	89.99	55.78	94.26	96.31
	F1 score	89.57	54.83	93.81	96.34
	AUC	91.68	55.91	95.07	96.48
	Specificity	92.16	65.74	95.43	98.74
Brain tumor MRI dataset	Test accuracy	97.63	78.93	92.49	99.54
	F1 score	96.25	76.20	91.83	99.56
	AUC	97.84	79.58	94.28	99.71
	Specificity	97.14	83.45	95.03	99.84
Chest cancer CT-scan dataset	Test accuracy	98.78	81.71	96.74	99.82
	F1 score	98.05	80.34	93.72	99.84
	AUC	99.47	81.92	98.03	99.90
	Specificity	99.12	88.24	97.91	99.91
Breast cancer microscopic biopsy image dataset	Test accuracy	91.85	80.35	92.47	96.76
	F1 score	89.30	80.11	90.34	96.29
	AUC	93.53	82.45	93.70	96.84
	Specificity	93.41	86.26	94.18	98.53
Breast cancer ultrasound image dataset	Test accuracy	96.43	85.61	93.43	98.75
	F1 score	96.18	83.57	93.18	97.61
	AUC	97.10	87.04	94.35	97.59
	Specificity	98.75	91.73	96.83	99.18

The results include test accuracy, specificity, F1 score, and area under the curve (AUC) statistical values.

**TABLE 7:** Results of Wilcoxon signed-ranked test.

Pairwise model comparison	*P*-value	Test outcome
Proposed model MNet-10 vs. VGG16	0.003	Significant
Proposed model MNet-10 vs. ResNet50	0.003	Significant
Proposed model MNet-10 vs. InceptionV3	0.003	Significant

MNet-10 is constructed and consists of a total of 10 layers and six weighted layers, and it is considered a shallow CNN model having about 10 million parameters; 143, 23, and 25 million parameters can be found in the state-of-the-art models VGG16, InceptionV3, and ResNet50, respectively, which are quite high for accommodating real-world data. Additionally, the ResNet-50 model shows a tendency to overfit on smaller datasets (82). Furthermore, models with a large number of trainable parameters take up a lot of time and resources in the training phase than shallow CNNs. Keeping all this in mind, the number of layers of the model is kept to a minimum to lessen the number of trainable parameters for better generalization even on a small dataset. With ablation studies, the lowest number of convolutional layers (four layers) is determined while maintaining optimal performance. Moreover, PReLU is utilized in MNet-10 rather than the traditional ReLU activation function for fast converge capabilities (83) and shows better overall performance. Faster convergence not only boosts the performance of a classifier but also contributes to minimizing computational complexity. Furthermore, small-sized convolutional kernels can extract more low-level textural information and small details resulting in better feature extraction from datasets containing tiny details. Hence, datasets with complex ROI (mammogram, chest X-ray, chest CT scan, Brain tumor MRI) benefit from the filter size of 3 × 3 of MNet-10. With this, overall performance boost is observed not only in small ROI datasets but also in datasets containing large ROI (Tympanic membrane and Skin cancer dataset), which adds to the generalization capabilities of the model across multiple datasets. In MNet-10, only one FC layer is used as multiple FC layers can introduce overfitting (84) for having dense connections (85). Furthermore, to address any potential overfitting issue, a dropout layer is added to randomly eliminate some connections of the FC layer (85) that are commonly used for feature generalization purposes.

Lastly, with our proposed MNet-10 model, stable performance can be observed across all the eight types of medical imaging modalities, with accuracies ranging between 96 and 99.6%, which adds to the effectiveness, consistency, and stability of the model.

## Discussion

Developing an optimal CNN classification model for medical image datasets of multiple diseases is the main goal of this research, and it has proven to be quite a challenging task. In this study, a robust shallow CNN model that can perform with optimal accuracy for all eight datasets even with the same parameters is developed. We consider that the most efficient way to achieve this is to develop the architecture using the mammogram dataset, which is regarded as one of the most challenging imaging modalities (86). For this goal, a number of ablation studies were conducted to generate the proposed MNet-10 model. The Ablation study has proven to be very effective, as it improved the classification capabilities of the proposed model from 89.55 to 97.34% (Figure 7) for the mammogram dataset. After developing the model with an optimal architecture, it is trained with the seven remaining datasets. It is also found that for the other datasets, the model is able to achieve a test accuracy above 96%. Therefore, our primary hypotheses become true that even without fine-tuning the parameters with other datasets, optimal performance can be achieved for all the datasets employing extensive experiments of ablation study using the most challenging imaging modality. Conducting intensive ablation studies on a complicated dataset such as those of mammograms made it possible for the model to learn even the smallest, complex, and hidden details, which led to better performance on datasets containing less complicated regions of interest (ear infection and skin cancer datasets).

As can be concluded from the literature review in Section “Dataset description,” although several experiments are conducted to build a model or preprocess a dataset, not enough experimentation regarding augmentation techniques is carried out. No study has explored a wide range of benchmark datasets of different diseases and imaging domains to evaluate the performance of a CNN model. Furthermore, which augmentation technique is more applicable for which imaging modality is a vital concern that needs more attention. As stated in Section “Data augmentation,” while working with grayscale images, hue and saturation cannot be applied as augmentation techniques despite being widely used. According to the PSNR values shown in Table 1, in RGB images, these techniques might drastically change significant pixel details and may produce a poor outcome. Moreover, the PSNR values indicate that new augmented images that are created using our chosen augmentation techniques do not change the pixel intensity level of the original image drastically compared to other photometric augmentation techniques.

To summarize, dealing with a limited number of training data with low computational complexity and training time, and a shallow CNN architecture can be an ideal approach. In this regard, a model should be developed in an effective way by employing an ablation study to set the parameters. However, in most cases, the annotated medical dataset is found to be too small to train a CNN model even with a shallow architecture. In these cases, data augmentation is performed to increase the volume of images introducing variations. According to our findings, regarding image augmentation techniques, various outcomes can be observed using the eight different datasets. In regard to the interpretation of medical images, applying an inappropriate algorithm for a particular dataset might lead to poor performance. Therefore, while dealing with medical images, before introducing any method including data augmentation, experimentation with the dataset should be carried out to identify the optimal approach. This study attempts to inaugurate the point that a shallow CNN model together with a suitable data augmentation technique can be the most ideal way to achieve optimal performance in medical image analysis. The result suggests that after developing the shallow architecture from the base model, the accuracy increases from 89.55 to 97.34% and that the number of parameters decreased from 66 to 10 million. With respect to the data augmentation technique, for all the modalities, the performance obtained from augmented datasets outperforms that from the non-augmented datasets. For all the non-augmented datasets, the accuracy was in the range of 66–88%. Depending on the optimal augmentation method on a particular dataset, the performance touches the peak across all the datasets resulting in a range of 96–99% accuracies. Moreover, an accuracy fluctuation of 3–7% is also observed across the modalities depending on the type of data augmentation technique. It can be concluded that data augmentation and a shallow network together aid in dealing with a limited number of images while shallow architecture impacts greatly on lowering the training time and time complexity.

## Data availability statement

Publicly available datasets were analyzed in this study. This data can be found here: Breast cancer Ultra sound image dataset. Kaggle repository: https://www.kaggle.com/aryashah2k/breastultrasound-images-dataset, CBIS-DDSM dataset. Kaggle repository: https://wiki.cancerimagingarchive.net/plugins/servlet/mobile?contentId=22516629#content/view/22516629, COVID-19 Radiography database. Kaggle repository: https://www.kaggle.com/tawsifurrahman/covid19-radiography-database, Skin cancer: Malignant vs. benign. Kaggle repository: https://www.kaggle.com/fanconic/skin-cancermalignant-vs-benign, Tympanic membrane dataset: https://figshare.com/articles/dataset/eardrum_zip/13648166/1, Brain tumor Classification (MRI). Kaggle repository: https://www.kaggle.com/sartajbhuvaji/brain-tumorclassification-mri, Break His 400X. Kaggle repository: https://www.kaggle.com/forderation/breakhis-400x, and Chest CT scan images Dataset. Kaggle repository: https://www.kaggle.com/mohamedhanyyy/chest-ctscan-images.

## Author contributions

SM, SA, and AR generated the main study concept and design. SM and AR carried out the study implementation, experiment, and statistical analysis with supervision and contributions from SA and MH. MH and KH contributed to the literature section and dataset formulation. SM and AR prepared and wrote the manuscript under the supervision of SA and MH and contribution of SP, MH, ZM, MJ, and AK. SA, MJ, and AK critically reviewed and edited the manuscript and gave final approval. All authors contributed to the research, manuscript writing, and approved the final version.
